# A Computational Pipeline for the Diagnosis of CVID Patients

**DOI:** 10.3389/fimmu.2019.02009

**Published:** 2019-08-30

**Authors:** Annelies Emmaneel, Delfien J. Bogaert, Sofie Van Gassen, Simon J. Tavernier, Melissa Dullaers, Filomeen Haerynck, Yvan Saeys

**Affiliations:** ^1^Department of Applied Mathematics, Computer Science and Statistics, Ghent University, Ghent, Belgium; ^2^Data Mining and Modeling for Biomedicine, VIB Center for Inflammation Research, Ghent, Belgium; ^3^Primary Immunodeficiency Research Lab, Center for Primary Immunodeficiency Ghent, Jeffrey Modell Diagnosis and Research Center, Ghent University Hospital, Ghent, Belgium; ^4^Department of Internal Medicine and Pediatrics, Ghent University, Ghent, Belgium; ^5^Department of Biomedical Molecular Biology, Ghent University, Ghent, Belgium; ^6^Unit of Molecular Signal Transduction in Inflammation, VIB Center for Inflammation Research, Ghent, Belgium; ^7^Ablynx, A Sanofi Company, Zwijnaarde, Belgium

**Keywords:** CVID, flow cytometry, FlowSOM, computational pipeline, PAD

## Abstract

Common variable immunodeficiency (CVID) is one of the most frequently diagnosed primary antibody deficiencies (PADs), a group of disorders characterized by a decrease in one or more immunoglobulin (sub)classes and/or impaired antibody responses caused by inborn defects in B cells in the absence of other major immune defects. CVID patients suffer from recurrent infections and disease-related, non-infectious, complications such as autoimmune manifestations, lymphoproliferation, and malignancies. A timely diagnosis is essential for optimal follow-up and treatment. However, CVID is by definition a diagnosis of exclusion, thereby covering a heterogeneous patient population and making it difficult to establish a definite diagnosis. To aid the diagnosis of CVID patients, and distinguish them from other PADs, we developed an automated machine learning pipeline which performs automated diagnosis based on flow cytometric immunophenotyping. Using this pipeline, we analyzed the immunophenotypic profile in a pediatric and adult cohort of 28 patients with CVID, 23 patients with idiopathic primary hypogammaglobulinemia, 21 patients with IgG subclass deficiency, six patients with isolated IgA deficiency, one patient with isolated IgM deficiency, and 100 unrelated healthy controls. Flow cytometry analysis is traditionally done by manual identification of the cell populations of interest. Yet, this approach has severe limitations including subjectivity of the manual gating and bias toward known populations. To overcome these limitations, we here propose an automated computational flow cytometry pipeline that successfully distinguishes CVID phenotypes from other PADs and healthy controls. Compared to the traditional, manual analysis, our pipeline is fully automated, performing automated quality control and data pre-processing, automated population identification (gating) and deriving features from these populations to build a machine learning classifier to distinguish CVID from other PADs and healthy controls. This results in a more reproducible flow cytometry analysis, and improves the diagnosis compared to manual analysis: our pipelines achieve on average a balanced accuracy score of 0.93 (±0.07), whereas using the manually extracted populations, an averaged balanced accuracy score of 0.72 (±0.23) is achieved.

## Introduction

Primary antibody deficiencies (PADs), the largest group of primary immune deficiency disorders, are characterized by markedly reduced serum levels of one or more immunoglobulin isotypes and/or inadequate antibody responses to specific antigens due to genetically determined defects in B cell development and/or function, without major impairments in other parts of the immune system. Common variable immunodeficiency (CVID) is one of the most prevalent PAD disorders, and defined as a marked decrease in serum immunoglobulin (Ig) G with a marked decrease in serum IgM and/or IgA, poor antibody responses to vaccination, and exclusion of secondary or other defined causes of hypogammaglobulinemia (1). As CVID is a diagnosis of exclusion, it encompasses a clinically and immunologically heterogeneous patient population with varying age of onset and severity. CVID patients typically have recurrent infections, mainly of the respiratory, and gastrointestinal tracts. In addition, CVID patients are prone to developing disease-related, non-infectious, complications due to immune dysregulation such as autoimmunity, polyclonal lymphoproliferation, granulomatous manifestations, and malignancy (1–3). Although various abnormalities in B and T cell subsets have been previously reported, the pathophysiological mechanisms of CVID are incompletely understood (1). In recent years, several disease genes for monogenic forms of CVID have been identified, but these only account for 2–10% of patients (4). Timely diagnosis of CVID remains an important challenge in clinical practice, where many other disease possibilities often have to be excluded before a definite diagnosis of CVID can be established (5, 6).

To aid the diagnosis of CVID patients, immunophenotyping by flow cytometry is often performed to obtain an overview of which immune cell populations are affected. Recent advances in multi-parameter flow cytometry allow the measurement of larger marker panels, so that increasingly detailed cell subsets can be identified (7–9).

Currently, flow cytometry data is typically analyzed manually by iteratively selecting cell populations on two-dimensional scatterplots. This manual approach is not only time-consuming, but also researcher-dependent and biased toward expected cell populations. In contrast, automated techniques may facilitate the analysis of larger marker panels by testing countless marker combinations, possibly identifying cell populations that might be indicative of disease status, which may have been overlooked during manual gating (10). In recent years, various computational techniques to analyze flow cytometry data have been developed (11). These techniques automate the different steps in the flow cytometry data analysis pipeline, making data analysis exactly reproducible. For example, pre-processing techniques such as FlowAI (12), and flowClean (13) can be used to perform data quality control. They automatically evaluate scatter and marker values over time and filter out regions that show abnormal behavior. To gain insight into the data structure, various techniques can be applied. Dimensionality reduction techniques such as PCA, t-SNE (14), or UMAP (15) perform dimensionality reduction, and project the high-dimensional cytometry data to a lower-dimensional (often two-dimensional) space, allowing a more comprehensive overview. On the other hand, automated population identification techniques also exist, that aim to group (cluster) similar cells into cell populations with similar phenotypes. To this end, many clustering algorithms have been developed (16), some of which also offer specific visualizations [e.g., FlowSOM (17) and Phenograph (18)]. Here, we develop a novel computational pipeline that combines several of these tools to help distinguish CVID patients from patients with other forms of PADs as well as healthy controls.

## Materials and Methods

### Study Cohort

The study cohort was described earlier in Bogaert et al. (9), in which extensive flow cytometric immunophenotyping was performed in patients with different forms of PADs, including CVID, and several control groups. From this cohort, we have reexamined the flow cytometry data from 28 CVID patients, 23 patients with idiopathic primary hypogammaglobulinemia, 21 patients with IgG subclass deficiency, six patients with isolated IgA deficiency, one patient with isolated IgM deficiency, and 100 healthy controls (HCs). CVID was defined as decreased [from hereon always meaning: at least two standard deviations (SD) below the age-adjusted mean according to the local lab reference values, measured at least twice] IgG, decreased IgA and/or IgM, and poor antibody responses to protein and/or polysaccharide vaccines (1). Idiopathic primary hypogammaglobulinemia was defined as decreased IgG, normal or decreased IgA and/or IgM, and good antibody responses to protein and/or polysaccharide vaccines. IgGSD was defined as decreased IgG2 and/or IgG3, normal total IgG and IgM, normal or decreased IgA, and good or poor antibody responses to protein and/or polysaccharide vaccines. Isolated IgA and IgM deficiencies were defined as an isolated decrease in IgA or IgM, respectively, normal IgG, and good antibody responses to protein and/or polysaccharide vaccines. Each patient was verified to fulfill the appropriate definition before enrollment in the study. Patients with other defined causes of antibody deficiency and/or profound T cell defects, as determined by the ESID registry criteria for CVID (http://esid.org/Working-Parties/Registry/Diagnosis-criteria), were excluded. For the current study, the patients with idiopathic primary hypogammaglobulinemia, IgG subclass deficiency, isolated IgA deficiency and isolated IgM deficiency were combined in one patient group from hereon referred to as “other PADs” (*n* = 51).

Three different marker panels were measured. The first panel focused on identifying the main cell populations in peripheral blood mononuclear cells (PBMCs), the second panel focused on B cell subsets, and the third panel focused on T cell subsets. A detailed overview of the marker panels can be found in Supplementary Table 1. The clinical variables gender, age, and diagnosis were collected from the patients' records. The patients were divided into eight age groups to adjust for age-dependent differences in white blood cell subsets [see Supplementary Table 2; (9, 19)]. Data were measured over 21 experiment days.

The study was approved by the ethical committee of Ghent University Hospital (2012/593). All reported subjects (or their parents in case of pediatric subjects) provided written informed consent for participation in the study, in accordance with the Helsinki Declaration of 1975.

### Computational Pipelines

We automated most steps of cytometry data analysis workflow, including quality control and data preprocessing, automated population detection, feature extraction, and predictive model building using machine learning methods to perform diagnosis. The scripts for the computational pipelines can be found on https://github.com/saeyslab/Computational_Pipeline_CVID.

#### Preprocessing and Quality Control

The fcs files were read into R, compensated with the compensation matrices determined in the previous study and transformed with the estimate Logicle function of the flowCore package. Cells with unreliable measurements (e.g., out of the detection range) were removed. Quality control was done with the FlowAI (12) package. Only the high quality measurements were selected for further processing. Additionally, only live single cells were used for further analysis, based on the manual pregating of the data.

Alongside the computational analysis, results were compared to the manual analysis performed in the original study [see Supplementary Figure 1; (9)]. The cell populations identified for panel 1 included B cells, CD4+ T cells, CD4- T cells, monocytes, natural killer T (NKT) cells, natural killer (NK) cells, basophils, dendritic cells (DCs), plasmacytoid DCs, and conventional DCs. For panel 2 this included IgD+CD27- naive B cells, IgD-CD27+ switched memory B cells (mem B cells), IgD+CD27+ marginal zone-like (MZ-like) B cells, IgD-CD27- B cells, CD24-CD38++ plasmablasts, CD24++CD38++ transitional (trans) B cells, CD21-CD38+ B cells, CD21low B cells, and CD19lowCD138+ plasma cells. The T cell panel 3 populations include CD31+RO-CD4+ T cells, CD31+RO-CD8+ T cells, CD4+ naive T cells, CD4+ effector memory T cells, CD4+ effector memory RA T cells, CD4+ central memory T cells, CD8+ central memory T cells, CD8+ effector memory T cells, CD8+ naive T cells, CD8+ effector memory RA T cells, gamma delta T cells, and regulatory T cells (Treg) cells.

#### Automated Population Identification and Feature Extraction

Cell populations were identified by FlowSOM, one of the best performing automated gating techniques identified in the benchmark by Weber et al. (16). FlowSOM uses a Self-Organizing Map (SOM) to group similar cells into fine-grained cell types, which are subsequently grouped into metaclusters (coarse-grained cell types) and visualized in a next step using a minimal spanning tree. FlowSOM trees were built separately for each panel with the FlowSOM R package. An aggregated file was created for each panel and contained 3.000.000 cells sampled from all the files for that panel. For panel 1, a FlowSOM model with a 10 ×10 grid and 14 metaclusters was created using the following markers: FSC-A, SSC-A, CD56, CD3, CD123, CD14, CD127, CD4, CD19, HLA-DR, iNKT/CD34, CD16, and CD11c. A FlowSOM model was created for panel 2 using a 10 × 10 grid and 18 metaclusters using the following markers: CD21, CD24, CD27, CD38, CD138, IgA, IgD, IgG, and IgM. The FlowSOM model created for panel 3 was also built with a 10 ×10 grid and 18 metaclusters using the following markers: CCR7, CXCR5, CD45RO, g/dTCR, FoxP3, CD278, CD8, CD31, and CD4. These were compared with the manual gating labels using the purity and F1-measure. Note that the purity is weighted for the number of cells belonging to a cluster.

Purity: $\frac{1}{N}\sum_{m\;\in M}\begin{array}{c} \max\\ d\;\in D \end{array}|m\cap d|$ (M: set of clusters, D: set of classes, N: number of cells)

F1-measure: $2^{*}\frac{precision\;\cdot recall}{precision+recall}$ Precision: $\frac{TP}{TP+FP}$ Recall: $\frac{TP}{TP+FN}$

(TP: True positives, FP: False positives, FN: False negatives)

For each panel, the following set of features were extracted to be used as input for the machine learning models in the next step: cell percentages for each file per cluster (percentages_clusters), per metacluster (percentages_metaclusters), and the cell percentages in the clusters compared to their respective metacluster (percentages_clusters_to_metaclusters). The median fluorescence intensities (MFIs) for all markers were also obtained for every cluster (MFI_cluster) and metacluster (MFI_metacluster). Zero-imputation was used for MFI values of clusters without cells. This resulted in a total of 1,696 features for panel 1, 1,162 features for panel 2 and 1,282 features for panel 3, yielding in total 4,140 features that can be used as input variables for the classifiers to perform automated diagnosing.

To eliminate effects linked to the aging of the immune system, a z-score was applied per age group on the extracted features of the clustering methods and on the features determined from the manual gating. The score was based on the mean (μ) and standard deviation (σ) of the healthy controls in each age group. All the individual values (x) for that age group and feature are normalized with the z-score: $Z_{age}=\;\frac{x_{age}-\;\mu_{age}}{\sigma_{age}}$

#### Automated Diagnosing Using Machine Learning

To perform automated diagnosis, we compared a number of machine learning models that aim to predict a patient's disease status from the features obtained from the automated gating. To this end, we explored two different types of classifiers: Random Forests, an ensemble based classifier based on a large combination of randomized decision trees, and Support Vector Machines (SVMs), a linear classifier that makes use of structural risk minimization to stimulate model generalization. Random Forest models were constructed using 500 decision trees, and SVM models were trained with a linear kernel function and C-parameter set to one. For each model type, two versions were trained. The first version formulated the problem as a three-class classification problem, distinguishing between CVID, other PADs and healthy patients. The second model version combined the other PADs and healthy control patients into a joint Non-CVID class vs. the CVID patients.

The full classification models were built with six different datasets consisting of the features extracted from the FlowSOM objects: percentages_clusters, percentages_metaclusters, percentages_clusters_to_metaclusters, Clusters (percentages_clusters + MFI_clusters), Meta_clusters (percentages_metaclusters + MFI_metaclusters), and total (percentages_clusters, percentages_metaclusters + MFI_clusters + MFI_metaclusters + percentages_clusters_to_metaclusters).

Model performance was measured using 21-fold cross-validation, leaving one experiment day out at the time. This ensures that the impact of batch effects on individual experiment days can be estimated accurately. The performance was assessed using the balanced accuracy measure due to the class imbalance for the CVID population compared to the other PAD and healthy control population.

Balanced accuracy: $\left(\frac{TP}{P}+\;\frac{TN}{N}\right)/2$ (TP: True positives, P: Positives, TN: True negatives, Negatives)

For each cross-validation run, one experiment day was left out to test generalization performance. Aggregated fcs files were created with the patient's fcs files missing for the left-out experiment day. FlowSOM objects were built for each aggregation file and age-group specific means and standard deviations for applying the z-scores were calculated at this point. Then all the files, including the left-out files, were mapped onto the FlowSOM object to extract their features and apply the z-scoring. Classification models were built with the features belonging to the train data (not the samples belonging to the left-out experiment day) and the test data was then used to predict their corresponding label.

#### Feature Selection

To get more insight into which features contribute most to model performance, and check whether removing unimportant features had a beneficial effect on classification performance, feature selection was performed. A feature selection method was applied to both the manual gating cell populations and the features derived from FlowSOM and is based on the feature selection step in Van Gassen et al. (20). For the classification models with two classes, Wilcoxon tests were calculated for every feature in the dataset based on the two labels (CVID vs. No-CVID). A Kruskal-Wallis test was performed for every feature if a classification model was built with CVID, other PADs, and HC labels. The *p*-values of these tests were then sorted and used for the feature selection step. The two features with the lowest *p*-values were selected, whereafter new features were iteratively added from the sorted list if the pairwise Pearson correlation between the selected features and the new candidate feature was lower than 0.2. These features were then used to build the classification models described in the previous section.

#### Classification Models Based on Manual Gating

In the original study, 47 features were extracted from the manual gating to describe the patients' immunophenotype. These values were normalized using the z-score and were also used as features to build the classification models. In the cross-validation step, the features calculated for the patients and healthy controls belonging to one experiment day were iteratively left out of the z-scoring and classification step and used as test data to predict their labels.

## Results

We compared the results of the automated pipeline based on automated quality control and population identification with FlowSOM to the results based on the manual gating. In a first step, we aimed to find out whether the population identification by FlowSOM corresponded to the manually gated populations. Subsequently, we evaluated the predictive power of features derived from both automated as well as manually gated populations in combination with machine learning models. Finally, we aimed to identify those features that seem most promising as biomarkers to diagnose CVID from other PADs and healthy controls.

### FlowSOM Accurately Identifies the Cell Populations

**Figure 1:**
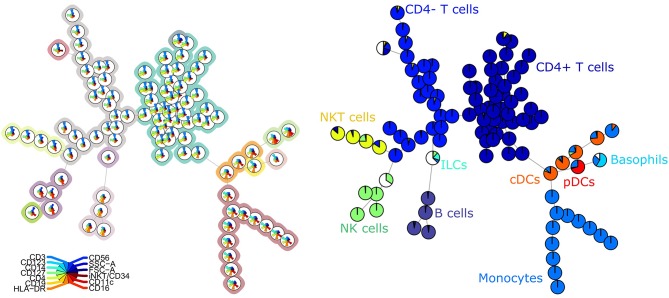
**Left:** FlowSOM tree for the PBMCs panel 1. The background coloring indicates the metaclustering. **Right:** FlowSOM tree were the cells from Healthy control PIDHC011 were mapped onto the original FlowSOM tree for panel 1. The colors of the nodes correspond to the manually gated labels.

The FlowSOM tree built for panel 1 coincided well with the manual labels, with an average purity per cluster of 0.94 (Figure 1). When grouping the clusters into metaclusters, the average purity was 0.78 and there was a clear correspondence with the manual populations (e.g., metacluster 10 corresponds with B cells). This translates into an F1-measure of 0.96 and is confirmed when looking at two-dimensional scatter plots corresponding with the manual gating strategy in Supplementary Figure 3.

**Figure 2:**
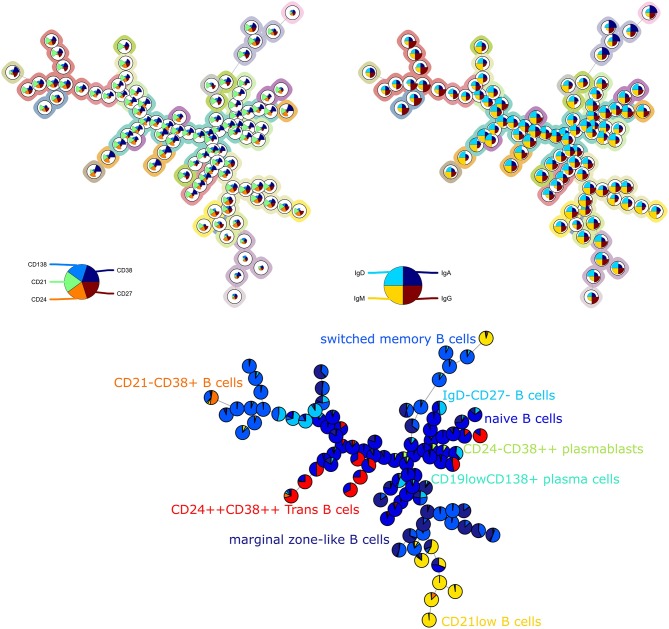
**Top left:** FlowSOM tree for the B cell subset panel 2 with expressed CD markers displayed. The background coloring indicates the metaclustering. **Top right:** FlowSOM tree with expression of immunoglobulins displayed. **Bottom:** FlowSOM tree where the cells from Healthy control PIDHC011 were mapped onto original FlowSOM tree for panel 2. The colors of the nodes correspond to the manually gated labels.

For the second panel, the average purity was 0.82 per cluster and 0.73 per metacluster, but the F1 measure only 0.60. This lower number is mainly due to a lower recall, which indicates that not all cells from the manually identified populations are captured together in one metacluster. When we noticed this discrepancy, we compared again the labeling on the tree (Figure 2) and the two-dimensional gating (Supplementary Figure 4). This inspection revealed that in the manual gating strategy, the cell populations are first determined based on their expression of CD markers and are later subdivided into smaller populations based on Ig expression. In contrast, the FlowSOM tree is largely split up into quadrants of immunoglobulins, and only then further split based on the CD markers.

**Figure 3:**
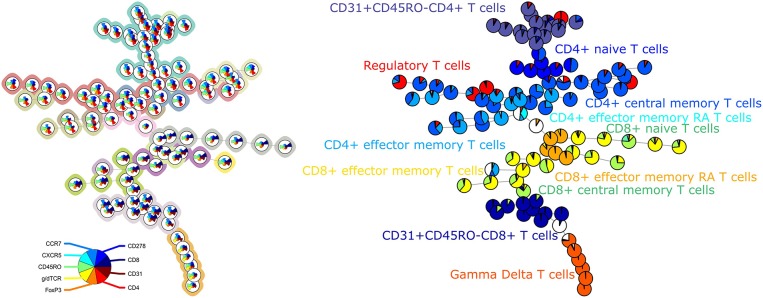
**Left**: FlowSOM tree for the T cell subset panel 3. The background coloring indicates the metaclustering. **Right**: FlowSOM tree were the cells from Healthy control PIDHC011 were mapped onto the original FlowSOM tree for panel 3. The colors of the nodes correspond to the manually gated labels.

The average purity per cluster and metacluster was 0.83 and 0.67, respectively, for panel 3 (Figure 3). The F-measure for this panel was 0.60. This lower purity and F-measure again indicate that not all the manually identified populations were captured together in one metacluster but the higher purity of the clusters indicates that the cells that are assigned to one cluster mostly belong to the same manually gated population. The purity of the clusters and metaclusters can also be visually inspected on Supplementary Figure 5.

The labeling of the clusters and metaclusters of all FlowSOM trees are depicted in Supplementary Figure 2.

### Machine Learning Models Accurately Diagnose CVID

**Figure 4:**
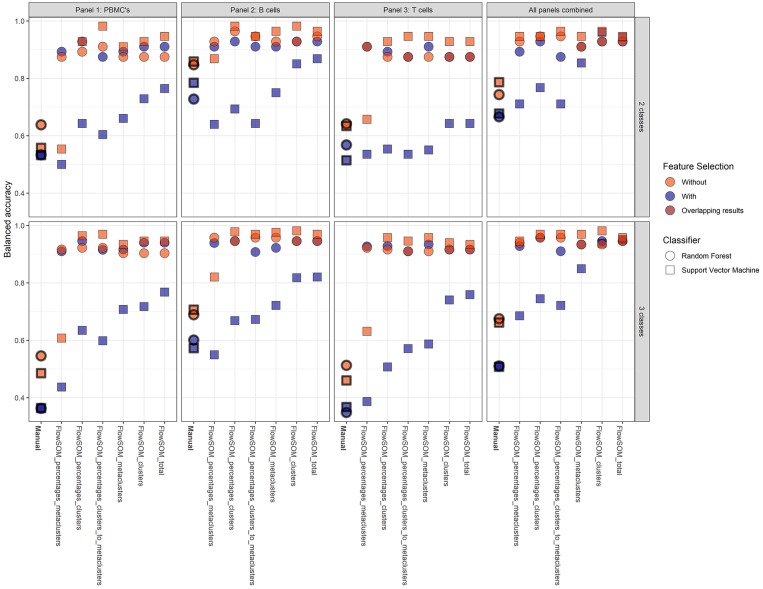
Overview of the balanced accuracy scores of the different classification models (performed with 21-fold cross-validation). Color indicates whether feature selection was applied, shape indicates which classification model was used. Overall, FlowSOM features can clearly improve on features extracted from the manual gating.

To assess the predictive power of the different classification models, a 21-fold cross-validation was performed for every classification model. The balanced accuracy was calculated in order to determine the predictive power of the extracted cell populations of FlowSOM or the manually extracted cell populations in combination with a classifier (random forest or SVM). An overview is given in Figure 4.

Overall, it is clear that the models with FlowSOM derived features are able to obtain higher scores than the pipelines using the features from the manual gating. The average balanced accuracy of all manual gating models for the prediction of the CVID and No-CVID class was 0.63 (±0.10) with feature selection and 0.79 (±0.15) without feature selection while the average balanced accuracy of all FlowSOM models was 0.80 (±0.13) and 0.91 (±0.07) with and without feature selection, respectively. For the three-class models the average accuracies was 0.45 (±0.10) with selection and 0.72 (±0.23) without selection for the manual gating features and 0.81 (±0.16) with selection and 0.93 (±0.0) without selection for the FlowSOM features. Several models only misclassified one patient, obtaining a balanced accuracy of 0.982.

In general, the SVM models gave more accurate results than the random forest classifiers if no feature selection step was used, except for a number of classification models built with the manually selected populations and the models using the FlowSOM metacluster percentages of the individual panels. If a feature selection step was used, all models with an SVM classifier performed worse than the random forests models that were built with the selected features. The decrease is smaller when the FlowSOM cluster MFIs and percentages are used together, or if all FlowSOM features are used for panel 2 or for the three panels combined. Feature selection before training the random forest models resulted most of the times in an equal or less result than the models where no initial feature selection step was performed. When using the features derived from the manually gated populations, feature selection always had a negative impact.

The models where features from panel 2 were used also had an overall better performance than the models based on features from panel 1, and were only slightly better in comparison with the models based on the features of the panels combined. When only the features of panel 3 were used, the performance decreased slightly for the FlowSOM features and greatly when the manually gated populations were used compared to the other two panels. When the information was combined of all three panels, the results generally increased compared to the individual panels. Only panel 2 generated some better results for particular models. The top accuracy was only reached for models built for panel 2 for the two-class model but the overall performance of the models is higher when three classes are classified.

In total, 112 models were built for both the two-class and three-class classification problems and built with either an SVM or random forest classifier, with one of the six different feature sets of FlowSOM or the manually selected populations and with or without a feature selection step. From all the possible two-class classification models, 79 individuals (out of 179) were misclassified at least once and nine patients were misclassified in more than 1/3th of the models (listed in Table 1). From all possible three-class classification models, 168 people were misclassified in the three-class classification models from which seven individuals were mislabeled in more than 1/3th of the models. This increase in mistakes between classification problems is due to added misclassifications between healthy controls and other PADs. For four of these ten patients, further follow-up with their physician indicated that they might have been misdiagnosed in our database.

**Table 1:** Overview of the most frequently misclassified patients.

	**Nr. misclassifications for two-class classification model**	**Nr. misclassifications three-class classification model**	**Remarks**
PID030 (CVID)	52	45	No explanation found yet.
PID040 (CVID)	70	73	Syndromic primary immunodeficiency initially presenting with CVID phenotype.
PID041 (CVID)	59	63	Syndromic primary immunodeficiency initially presenting with CVID phenotype. First degree family member of PID040.
PID043 (Other PAD)	0	64	Early loss to follow-up, no information on progression of disease phenotype.
PID053 (CVID)	111	110	No explanation found yet.
PID054 (CVID)	104	100	No explanation found yet. First degree family member of PID053.
PID055 (CVID)	78	70	Presumably secondary CVID after autoimmune—induced subacute liver failure with need of liver transplantation.
PID060 (CVID)	42	35	No explanation found yet.
PID257 (CVID)	38	29	No explanation found yet.
PID285 (CVID)	39	33	No explanation found yet.

*All the patients listed in the first column were misclassified by the models in more than 1/3 of the 112 possible model combinations (built with or without a feature selection, with either an SVM or random forest classifier, with one of six different feature sets of FlowSOM or the manually selected populations). The second column depicts the results for all two-class models while the third column shows the results for the three-class models. In the last column, remarks are listed as possible explanations for the frequent misclassification. The red color of a number indicates that that patient was not misclassified in more than 1/3th of the 112 models*.

### Visual Exploration of the Immune State Space Allows for Visual Separation of CVID Patients

**Figure 5:**
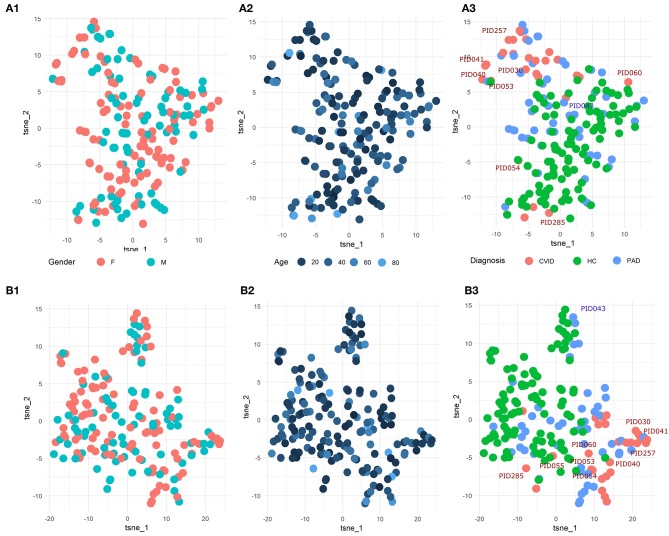
**(A1-3)** t-SNE result of the patient population with manually gated cell populations of the most important features determined by the SVM model for the three panels individually. **(B1-3)** t-SNE result of the patient population with the total features of FlowSOM of the most important features determined by the SVM model for the three panels individually. The perplexity for both t-SNEs was set to 15. A z-score was applied on the used features first to eliminate age-linked immune changes.

To explore the underlying structure of the patient population, dimensionality reduction using t-SNE was performed on the three features with the highest importance scores in the SVM models built on either the manual gating results for the three panels individually or the total feature set extracted from FlowSOM for both panels individually (Figure 5). For both models, there is no grouping visible based on age or gender.

Looking at diagnosis, there is a grouping of healthy controls for both the model built on the manual features and for the model built on the FlowSOM features. Most CVID patients also seem to group together for both models with the exception of some. The other PAD patients however seem to be spread across the healthy controls and the CVID patients.

### Most Important Features in SVM Classification Identify Relevant Populations for CVID Diagnosis

**Figure 6:**
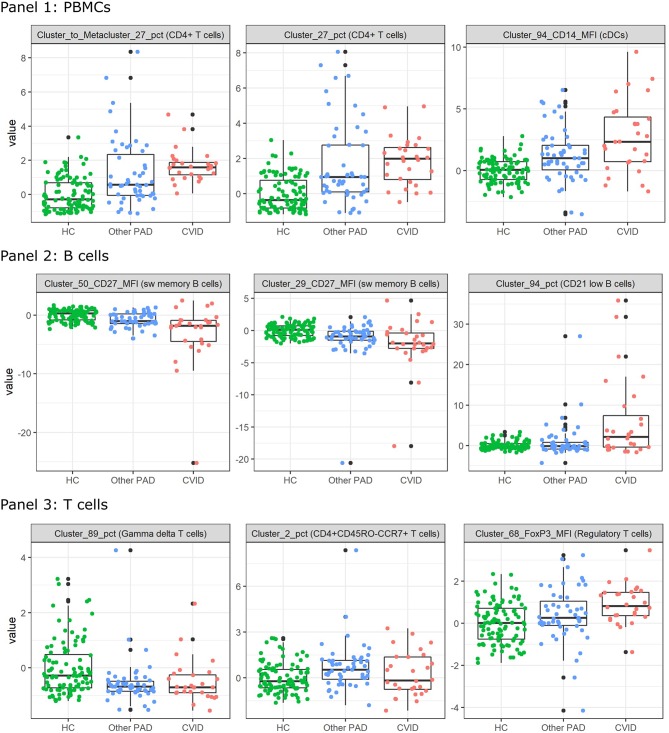
Boxplots calculated for the most important features in the support vector machines built for the three-way classification of all FlowSOM features for either panel 1, 2, or 3. The colored points indicate the values on which the boxplots were built. A z-score was applied on the used features first to eliminate age-linked immune changes.

In order to find populations that could play a role in the identification of CVID patients, importance scores were ranked from the SVM result in the three-class classification models for panels 1-3 individually. These are the same features as in the dimensionality exploration section. The expression of the top three features from the models that were built with all the FlowSOM features (i.e., the MFIs and percentages of clusters and metaclusters and the percentages of clusters compared to the metaclusters) were compared across the three different classes and are visualized in Figure 6 for the three panels.

For all features of the first panel, an increasing trend is visible of the feature values for the CVID patients. These results indicate an increase in a certain CD4+ T cell population compared to the entire CD4+ T cell population and an increase of CD14 expression on a certain population of cDCs. For the second panel there seems to be a decrease of CD27 expression for a particular switched memory B cell population with IgG, CD24 and CD21 expression. A similar trend is visible for another switched memory B cell population where no immunoglobulins but the same CD markers are expressed. The last feature of panel 2 indicates an increase of a CD21low B cell population in CVID patients compared to the other two classes. Three different types of T cells were selected for panel 3. The first population consists of a specific gamma delta T cell population that appears to have decreased in the other PAD and CVID patients. There seems to be a decrease of the CD4+ naive T cells (CD45RO-CCR7+) in the other PAD patients opposed to the healthy controls but this decrease is not greatly extended to the CVID patients. The last feature indicates an increase in MFI expression of FoxP3 for a certain regulatory T cell population.

### Feature Selection Identifies Relevant Population for CVID Diagnosis

Although the feature selection steps did not seem to improve the models, the selected features with the lowest *p*-values still seem to be relevant in the identification of the CVID patients.

The number of selected FlowSOM features ranged from 2 to 43 features with a median of 13 features while the number of the selected manual populations ranged from 4 to 15 features with a median of seven features.

**Figure 7:**
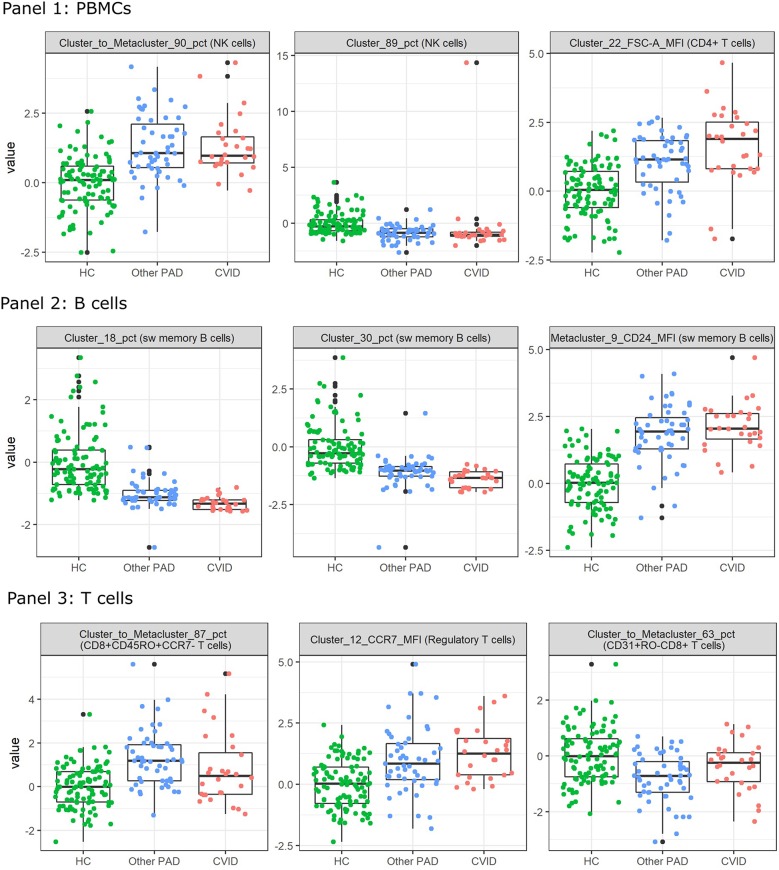
Boxplots calculated for the features selected by the feature selection step in the three-class classification model calculated on all FlowSOM features for either panel 1, 2, or 3. This feature selection step is performed before the classification step in the automated models. The first three features selected with the lowest *p*-value are displayed for both panels. The colored points indicate the values on which the boxplots were built. A z-score was applied on the used features first to eliminate age-linked immune changes.

To inspect these features for the three-class classification problem, Kruskal Wallis tests were performed on all the FlowSOM features. The two features with the lowest *p*-values and the next feature with the lowest *p*-value and with a low correlation with the first two features were selected. They all had a value smaller than 1.10–10 and are depicted in Figure 7. The features selected for panel 1 indicate an increase of a certain NK cell population compared to the entire NK cell population for the other PAD and CVID patients. For another NK cell population however, there seems to be a decrease in cell counts compared to all immune cells. The difference between the two populations is the expression of CD56. Cluster 89 shows a very low expression of the marker. The final selected feature of panel 1 suggests that for cluster 22, cells (labeled as CD4+ T cells) have a larger cell size due to higher FSC-A values in the other PAD and CVID patients. Upon further inspection, we confirmed that this significant increase was not only the case for cluster 22, but also for the FSC-A MFI of most CD4+ T cell clusters and the metacluster 1 which represents the CD4+ T cells. Unfortunately this could not be confirmed in panel 3 due to altered scattered values because of the fixation step necessary for the use of the intracellular marker FoxP3.

For panel 2, a significant decrease is present in two certain switched memory B cell populations, with IgG, CD38, and CD21 expressed, compared to the healthy controls and the other PAD patients. A second switched memory B cell population seems to be increased in CVID patients compared to the other two classes that expresses CD21, IgA, and CD24.

In panel 3, an increase can be found for a certain population of CD8+ effector memory T cells (CD45RO+CCR7-) compared to the other populations in its metacluster in the other PAD patients. This increase toward the healthy controls is also present in the CVID patients but not as pronounced. The MFI however of CCR7 in a certain cluster of regulatory T cells is increased gradually from the healthy controls to the other PADs and the CVID patients. The third important feature of the SVM model is again a cluster to metacluster ratio where a great decrease is noticed in the other PADs compared to the healthy controls and a smaller decrease in the CVID patients of a certain CD31+CD45RO-CD8+ T cell population.

### Diagnosis of a New Patient Is Much Faster by Automated Classification Than by Manual Analysis

A big advantage of using automated models during the diagnostic process of patients is that the computational time is much less than the time necessary to manually gate every population in 2D plots. It took our model 2.54 min in total to load the necessary objects to make a prediction, to map the fcs file of a randomly chosen patient on the FlowSOM tree created for panel 1, to extract all possible features (percentages and MFIs), to apply the z-score to these features and to make a prediction with the SVM of a three-class model built on all possible FlowSOM features. When the classification would be done with a manual gating strategy it would take at least 15 min to gate the entire manual gating strategy and classify the patient based on the known CVID criteria. The classifier built on these gated populations, as performed in this study, still uses the populations that have to be gated first which occupies most of the work and time. This means that the usage of a full computational model is much faster than performing classification based on manually placed gates.

## Discussion

In this study, the efficiency and accuracy of different models built with varying FlowSOM feature sets or manually gated cell populations were compared in their ability to identify CVID patients, patients with other PADs and healthy controls.

Both types of automated techniques can be used to diagnose CVID but models built on FlowSOM features proved to be faster and more accurate than models built on manually gated cell populations. Even when the number of FlowSOM features dropped lower than the total number of manually gated populations due to feature selection, the random forest models still obtained better results than the models built with all manual features.

The FlowSOM models accurately represented the manually gated cell populations and captured more detail allowing for better classification results with either an SVM or a random forest as classifier. This is confirmed by the features extracted from the FlowSOM tree of panel 2. Although the structure of the tree of this panel does not coincide with the manual gating strategy since it first splits up the immunoglobulins instead of the CD markers, the classification results of the extracted features from this tree still managed to obtain better accuracies in predicting the class labels.

The comparison between these classifiers showed that models built with an SVM outperformed the models with a random forest when no feature selection was applied. However, when this selection limited the number of features, the accuracy of the SVM models dropped below the accuracy of the random forest models.

Adding this feature selection step, in order to remove unimportant features, did not increase the accuracy of the models. For the models built on the manually gated populations, this is explained by the limited number of cell populations that were gated. These populations were specifically selected according to commonly used gating guidelines in order to investigate CVID patients, meaning that filtering these cell populations resulted in a loss of information needed to classify the patients correctly. The fact that CVID is an immunologically heterogeneous disease could explain the accuracy drop for all models built on FlowSOM features. This would indicate that there are many differential alterations in the immune system in CVID patients, other PAD patients and healthy controls and that the distinction between these groups cannot be made based on only a few of them. By performing a feature selection step, too much valuable information would be lost. The heterogeneity of the disease is confirmed by the selection of other features that were considered as most important in the classification step for the different models and by selecting other features during the feature selection step than those that were most important in the SVM or random forest models.

Though it did not seem that feature selection added an advantage in increasing accuracy of the predictions, it still gave valuable insights into the CVID phenotype next to the features that were ranked as most important in the SVM classification. Most of these insights are confirmed in the original study of Bogaert et al. (9) where they also noticed a general decrease of switched memory B cell populations, a decrease of switched memory B cells that express IgG and an increase of CD21low B cells in CVID patients, a decrease in CD4+ naïve T cells and a decrease in NK cells in CVID patients and in patients with other PADs. However, two features, concerning CD4+ T cells, seem to contradict results from the original study. The original results showed a decrease of the general CD4+ T cell population while these features indicates an increase for one CD4+ T cell population in CVID patients and in patients with other PADs. Nevertheless, this is merely an indication of only one CD4+ T cell population that seems to be increased as opposed to the entire CD4+ T cell population.

The advantage of using a FlowSOM model allowed for a deeper insight into these results and delivered a more specific marker profile of the cells (i.e., the decreasing szitched memory B cell percentages for clusters 18 and 30 expressed next to IgG also CD38 and CD21). This also allowed for the comparison in MFIs of markers between cell populations which lead to a certain sw memory B cell population that expressed CD21, CD24, and IgA. For this cell population in CVID and other PAD patients, the CD24 expression was increased. Other examples of the specificity of FlowSOM are the features that either showed the increase of FoxP3 for a certain regulatory T cell population in other PAD but mostly in CVID patients and the feature that showed the increase of CCR7 for another regulatory T cell population.

Multiple features highlighted in this study have not yet been discussed in literature. They concern the increase of the FSC-A marker in the CD4+ T cell population in CVID and other PAD patients, the increase of the CD14 markers for a certain conventional dendritic cell population, the increase in CD8+ effector memory T cells and in CD8+CD45RO-CD31+ T cells and the decrease of certain gamma delta T cells in the other PAD and CVID patients.

There are two features that did not seem to be related to the CVID or other PAD phenotypes that indicate a decrease of the CD27 marker for two switched memory B cell populations. This is explained by the mapping of IgD-CD27- cells instead of switched memory B cells on these clusters of the FlowSOM model for the CVID and other PAD patients.

Another important aspect of the classification models concerns the frequently misdiagnosed patients. Although the best model could predict almost all CVID patients, mistakes were still made. It was shown that these mistakes could be valid and that the follow-up of these patients can give valuable insights into the model. It seemed that two misclassified patients were wrongly diagnosed and that secondary complications have a marked influence on the classification of the patients. However, most of these patients with secondary complications are still correctly diagnosed in the best performing models.

The final conclusion of this study tells us that the use of a classification model built on FlowSOM features would be a quick, accurate and useful tool in the diagnosis of patients with CVID. This, however, should still be confirmed in a larger cohort with a more generalized marker panel in order to integrate the classification models in the daily diagnostic procedures.

## Data Availability

The datasets generated for this study are available on request to the corresponding author.

## Ethics Statement

This research was approved by the ethical committee of Ghent University Hospital (2012/593). All reported subjects (or their parents in the case of pediatric subjects) provided written informed consent to participation in the study, in accordance with the Helsinki Declaration of 1975.

## Author Contributions

AE, DB, SV, FH, ST, and YS all contributed to writing the manuscript. AE generated the results of the manuscript under supervision of SV and YS. DB and MD set up the original study and provided the manually gated labels and all fcs files and clinical information for this study. FH, ST, and DB explained the biology of the disease and linked the outcome of the computational models to the clinical phenotypes of the patients.

### Conflict of Interest Statement

The authors declare that the research was conducted in the absence of any commercial or financial relationships that could be construed as a potential conflict of interest. The reviewer JS and handling editor declared their shared affiliation.

## Supplementary Material

Supplementary Figure 1 Manual gating strategies. The manual gated strategies used to determine the manually selected cell populations is depicted in the figure for panel 1, panel 2, and panel 3.

Supplementary Figure 2 Overview of clustering and meta-clustering labels of FLowSOM trees. Cluster- and meta-clusterlabels were created by building the FlowSOM models for the three panels. These clusterlabels (left) and meta-clusterlabels (right) are used for labeling in the feature extraction step. **(A)** FlowSOM model created for the panel 1 with focus on Peripheral Blood Mononuclear cells. **(B)** FlowSOM model created for the panel 2 with focus on B cell subsets. **(C)** FlowSOM model created for the panel 3 with focus on T cell subsets.

Supplementary Figure 3 Comparison of metaclusters with manual gating panel 1. The manual gated plots are depicted on the first row for PIDHC011 and correspond to the same marker combinations visualized in Supplementary Figure 1. The following rows visualize 2D scatterplots of the same marker combinations of the manual gates in the first row for each metacluster. 100,000 cells were randomly sampled from the FlowSOM tree from panel 1. The background cells are colored black while those of the selected metacluster are plotted in color.

Supplementary Figure 4 Comparison of metaclusters with manual gating panel 2. The manual gated plots are depicted on the first row for PIDHC011 and correspond to the same marker combinations visualized in Supplementary Figure 1. The following rows visualize 2D scatterplots of the same marker combinations of the manual gates in the first row for each metacluster. 100,000 cells were randomly sampled from the FlowSOM tree from panel 2. The background cells are colored black while those of the selected metacluster are plotted in color.

Supplementary Figure 5 Comparison of metaclusters with manual gating panel 3. The manual gated plots are depicted on the first row for PIDHC011 and correspond to the same marker combinations visualized in Supplementary Figure 1. The following rows visualize 2D scatterplots of the same marker combinations of the manual gates in the first row for each metacluster. 100,000 cells were randomly sampled from the FlowSOM tree from panel 1. The background cells are colored black while those of the selected metacluster are plotted in color.
