# Supplementary material for: A Computational Pipeline for the Diagnosis of CVID Patients
Source: Front Immunol. 2019 Aug 30;10:2009. doi: 10.3389/fimmu.2019.02009 (PMC6730493; doi:10.3389/fimmu.2019.02009)
Supplement: Supplementary file 1 [file Table_1.DOCX]

Supplementary table 1: Overview of stainings for the two studied panels. Panel 1 represents a more general staining for white blood cells, while panel 2 contains stainings to distinguish different B cell subsets. The third panel represents stainings that can distinguish T cell subsets. The left column shows the used fluorophores while the other columns indicate their target molecules.

|  | **Panel 1**  **(PBMCs)** | **Panel 2**  **(B cells)** | **Panel 3**  **(T cells)** |
| --- | --- | --- | --- |
| **FITC** | CD56 | CD27 | CD8 |
| **PerCP-Cy5.5** | CD3 | CD24 | ICOS |
| **PE** | CD123 | IgA | CCR7 |
| **PE-CF594** | CD14 | CD21 | CXCR5 biotin  SA PE-CF594 |
| **PE-Cy5** | CD127 | IgM | CD45RO |
| **PE-Cy7** | CD4 | CD38 | g/d TCR |
| **APC** | CD19 | IgG | FoxP3 |
| **AF700** | HLA-DR | CD20 | CD3 |
| **APC-Cy7** | CD16 | CD19 | CD25 |
| **Pacific Blue** | CD11c | CD138 | CD4 |
| **AmCyan** | live/dead | live/dead | live/dead |
| **BV605** | iNKT biotin  CD34 biotin  SA BV605 | IgD | CD31 |

Supplementary Table 2: Overview of spread of patients over different age groups used for z-scoring. The last row indicates the number of patients belonging to a certain group.

| ***Age group*** | **3** | **4** | **5** | **6** | **7** | **8** | **9** | **10** |
| --- | --- | --- | --- | --- | --- | --- | --- | --- |
| ***Age*** | 4.1-  6.0y | 6.1-  10.0y | 10.1-  16.0y | 16.1-  20.0y | 20.1-  30.0y | 30.1-4  0.0y | 40.1-  50.0y | 50.1-  90.0y |
| ***Nr. Patients*** | 3 | 13 | 32 | 18 | 27 | 29 | 22 | 35 |
