# Supplementary figures and images for: A Computational Pipeline for the Diagnosis of CVID Patients
Source: Front Immunol. 2019 Aug 30;10:2009. doi: 10.3389/fimmu.2019.02009 (PMC6730493; doi:10.3389/fimmu.2019.02009)

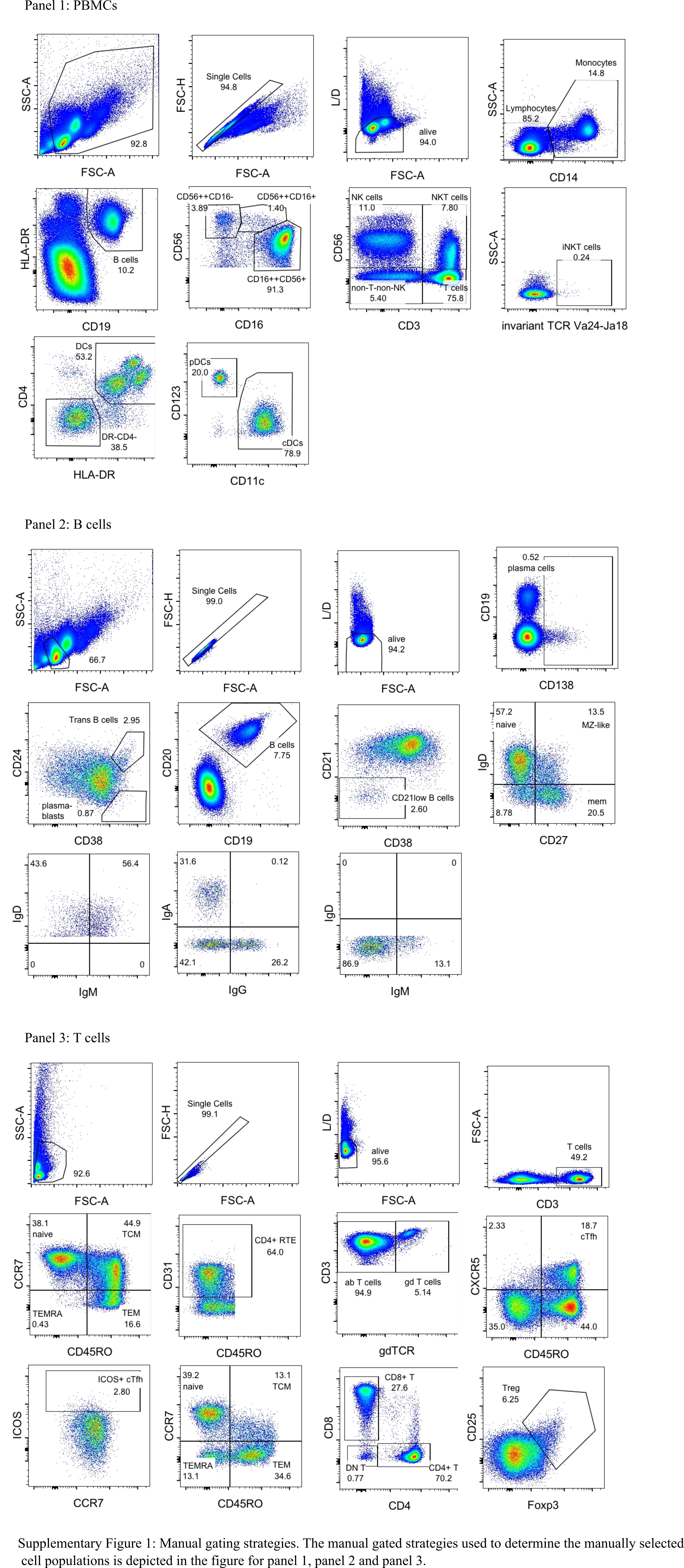

Supplement: Supplementary Figure 1 — Manual gating strategies. The manual gated strategies used to determine the manually selected cell populations is depicted in the figure for panel 1, panel 2, and panel 3. [file Image_1.PNG]

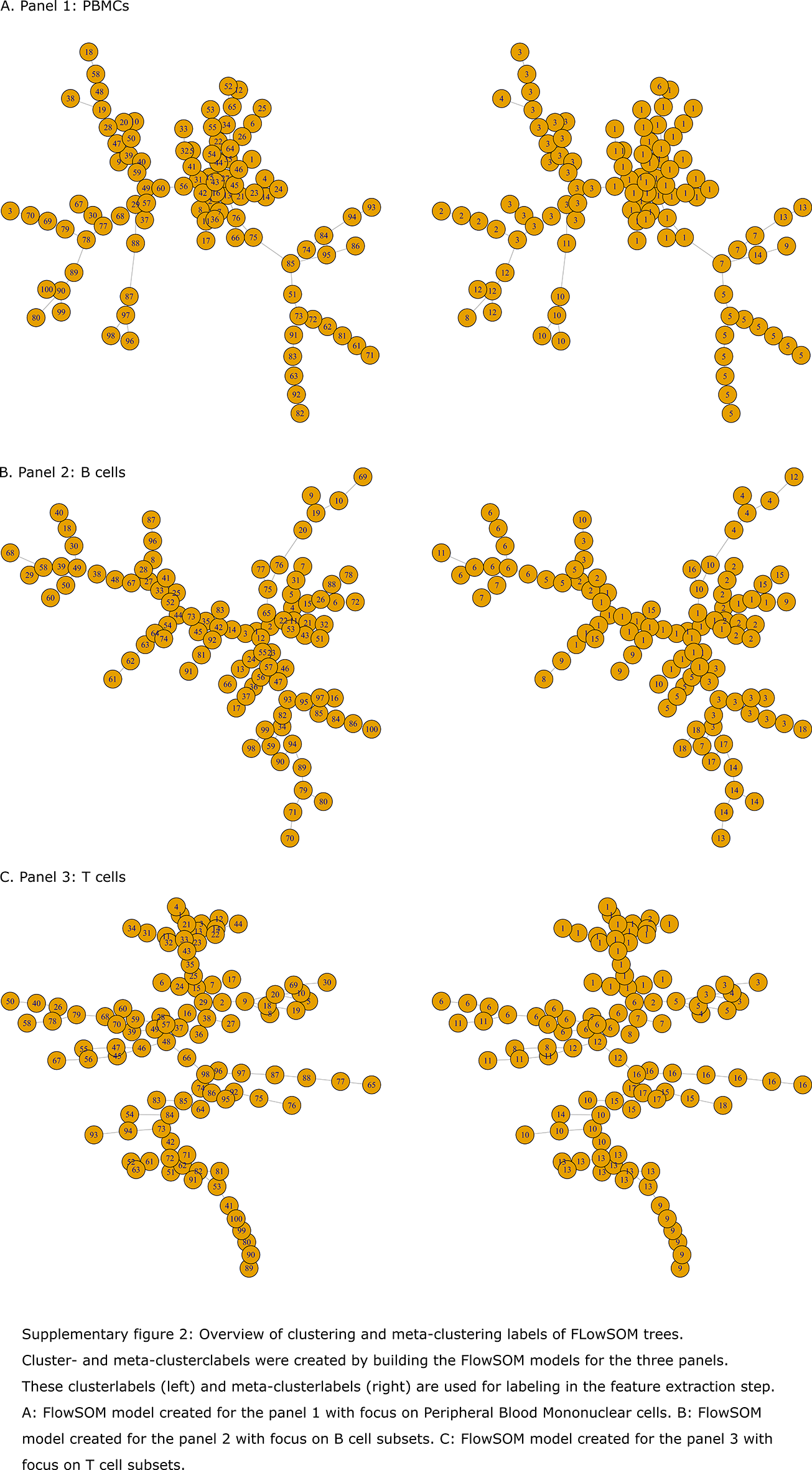

Supplement: Supplementary Figure 2 — Overview of clustering and meta-clustering labels of FLowSOM trees. Cluster- and meta-clusterlabels were created by building the FlowSOM models for the three panels. These clusterlabels (left) and meta-clusterlabels (right) are used for labeling in the feature extraction step. (A) FlowSOM model created for the panel 1 with focus on Peripheral Blood Mononuclear cells. (B) FlowSOM model created for the panel 2 with focus on B cell subsets. (C) FlowSOM model created for the panel 3 with focus on T cell subsets. [file Image_2.PNG]

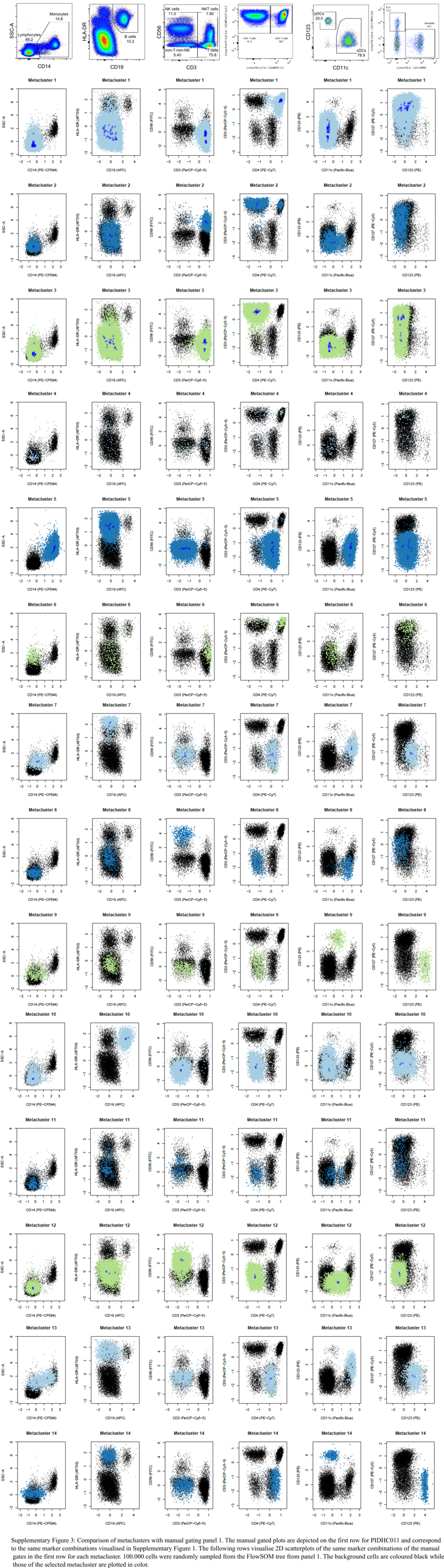

Supplement: Supplementary Figure 3 — Comparison of metaclusters with manual gating panel 1. The manual gated plots are depicted on the first row for PIDHC011 and correspond to the same marker combinations visualized in Supplementary Figure 1. The following rows visualize 2D scatterplots of the same marker combinations of the manual gates in the first row for each metacluster. 100,000 cells were randomly sampled from the FlowSOM tree from panel 1. The background cells are colored black while those of the selected metacluster are plotted in color. [file Image_3.PNG]

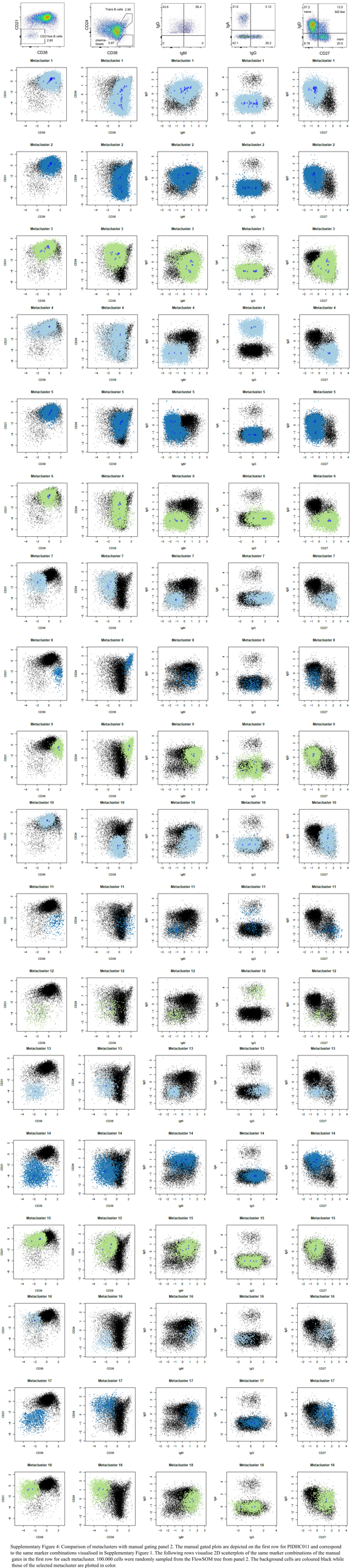

Supplement: Supplementary Figure 4 — Comparison of metaclusters with manual gating panel 2. The manual gated plots are depicted on the first row for PIDHC011 and correspond to the same marker combinations visualized in Supplementary Figure 1. The following rows visualize 2D scatterplots of the same marker combinations of the manual gates in the first row for each metacluster. 100,000 cells were randomly sampled from the FlowSOM tree from panel 2. The background cells are colored black while those of the selected metacluster are plotted in color. [file Image_4.PNG]

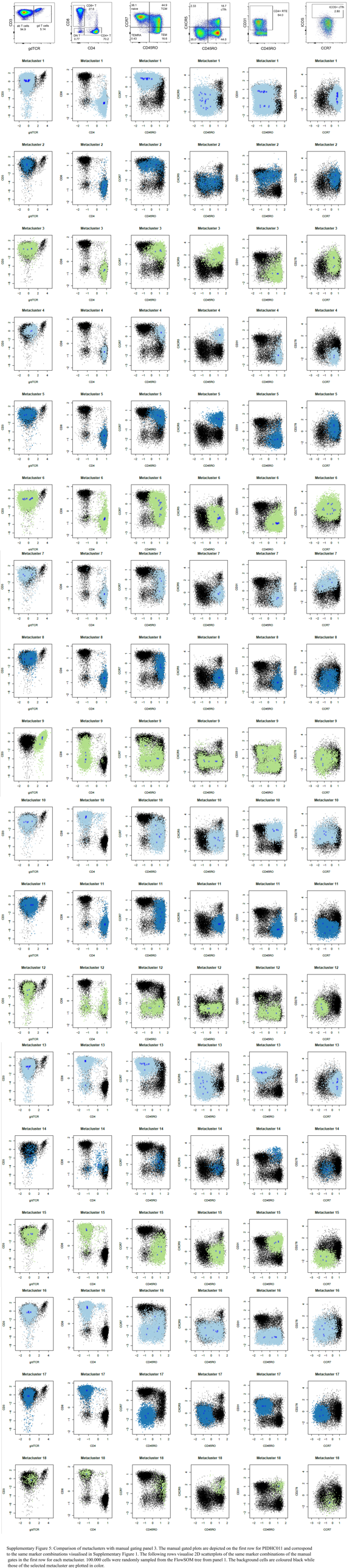

Supplement: Supplementary Figure 5 — Comparison of metaclusters with manual gating panel 3. The manual gated plots are depicted on the first row for PIDHC011 and correspond to the same marker combinations visualized in Supplementary Figure 1. The following rows visualize 2D scatterplots of the same marker combinations of the manual gates in the first row for each metacluster. 100,000 cells were randomly sampled from the FlowSOM tree from panel 1. The background cells are colored black while those of the selected metacluster are plotted in color. [file Image_5.PNG]
